# Cognitive-Postural Multitasking Training in Older Adults – Effects of Input-Output Modality Mappings on Cognitive Performance and Postural Control

**DOI:** 10.5334/joc.146

**Published:** 2021-03-10

**Authors:** Markus Brahms, Stephan Heinzel, Michael Rapp, Volker Reisner, Gunnar Wahmkow, Jérôme Rimpel, Gesche Schauenburg, Christine Stelzel, Urs Granacher

**Affiliations:** 1 Division of Training and Movement Sciences, Research Focus Cognition Sciences, University of Potsdam, Potsdam, Germany; 2 Clinical Psychology and Psychotherapy, Freie Universität Berlin, Berlin, Germany; 3 University of Potsdam, Research Focus Cognitive Sciences, Division of Social and Preventive Medicine, Potsdam, Germany; 4 Max Planck Institute for Human Cognitive and Brain Sciences, Leipzig, Germany; 5 International Psychoanalytic University, Germany

**Keywords:** aging, dual task, motor control, modality compatibility, balance training

## Abstract

Older adults exhibit impaired cognitive and balance performance, particularly under multi-task conditions, which can be improved through training. Compatibility of modality mappings in cognitive tasks (i.e., match between stimulus modality and anticipated sensory effects of motor responses), modulates physical and cognitive dual-task costs. However, the effects of modality specific training programs have not been evaluated yet. Here, we tested the effects of cognitive-postural multi-tasking training on the ability to coordinate task mappings under high postural demands in healthy older adults. Twenty-one adults aged 65–85 years were assigned to one of two groups. While group 1 performed cognitive-postural triple-task training with compatible modality mappings (i.e., visual-manual and auditory-vocal dual n-back tasks), group 2 performed the same tasks with incompatible modality mappings (i.e., visual-vocal and auditory-manual n-back tasks). Throughout the 6-weeks balance training intervention, working-memory load was gradually increased while base-of-support was reduced. Before training (T0), after a 6-week passive control period (T1), and immediately after the intervention (T2), participants performed spatial dual one-back tasks in semi-tandem stance position. Our results indicate improved working-memory performance and reduced dual-task costs for both groups after the passive control period, but no training-specific performance gains. Furthermore, balance performance did not improve in response to training. Notably, the cohort demonstrated meaningful interindividual variability in training responses. Our findings raise questions about practice effects and age-related heterogeneity of training responses following cognitive-motor training. Following multi-modal balance training, neither compatible nor incompatible modality mappings had an impact on the observed outcomes.

## Introduction

Age-related decrements in cognitive-motor multitasking are well documented (Granacher, Bridenbaugh, Muehlbauer, Wehrle, & Kressig, 2011; Granacher, Mühlbauer, Bridenbaugh, Wehrle, & Kressig, 2010; Rapp, Krampe, & Baltes, 2006; Voelcker-Rehage, Stronge, & Alberts, 2006; Woollacott & Shumway-Cook, 2002) and have high relevance for everyday activities and critical outcomes such as falls (Beauchet et al., 2009; Lajoie & Gallagher, 2004). Cognitive-motor multitasking decline in old age is indicated by increased dual-task costs for motor demands such as standing or walking while concurrently performing complex cognitive tasks (e.g., arithmetics) (Beauchet, Dubost, Aminian, Gonthier, & Kressig, 2005), having a conversation (Ayers, Tow, Holtzer, & Verghese, 2014; Beauchet et al., 2009), or performing more basic concurrent attentional or cognitive experimental paradigms (Al-Yahya et al., 2011). Depending on the processing requirements of the cognitive task, dual-task costs (DTC) are present in cognitive performance, motor performance, or both, suggesting a dynamic interplay between both domains. Different factors may contribute to a shift in task prioritization and associated domain-specific dual-task costs – amongst others, this may be related to task load (Bernard-Demanze, Dumitrescu, Jimeno, Borel, & Lacour, 2009; Maclean, Brown, Khadra, & Astell, 2017; Teasdale, Bard, LaRue, & Fleury, 1993).

Aging is associated with changes in cognitive and motor function, such as a decline in working memory capacity (Heinzel et al., 2014; Sander, Lindenberger, & Werkle-Bergner, 2012) and postural stability (Choy, Brauer, & Nitz, 2003; Gill et al., 2001; Granacher et al., 2011). Greater resource demands on a cortical level likely contribute to cognitive-motor interference (Jacobs & Horak, 2007; Mihara, Miyai, Hatakenaka, Kubota, & Sakoda, 2008). Alternatively or subsidiary to these domain-specific changes, the reported decrements might be related to a decline in higher order cognitive control functions involved in the coordination of concurrent task performance within or between domains (Meyer & Kieras, 1997; Walshe, Patterson, Commins, & Roche, 2015). Recently, Stelzel and colleagues (2017) provided evidence for an interplay of all three factors, i.e. impaired posture, working memory decrements and decrements in cognitive control processes. In that study, we systematically manipulated working memory load by comparing cognitive single vs. dual one-back working-memory tasks and control demands in a continuous cognitive working-memory task, performed concurrently with a postural task on a force plate. The degree of control demands was manipulated using the compatibility of input-output modality pairings of the component tasks (Hazeltine, Ruthruff, & Remington, 2006; Stelzel, Schumacher, Schubert, & D’Esposito, 2006). Previous studies have consistently shown increased cognitive performance costs for modality incompatible mappings (e.g., visual-vocal and auditory-manual) compared to modality compatible mappings (e.g., visual-manual and auditory-vocal). These modality-specific costs for correctly assigning stimuli to required responses were shown in dual-task paradigms involving task-set switching or concurrent task performance (Fintor, Stephan, & Koch, 2018; Göthe, Oberauer, & Kliegl, 2016; Stephan & Koch, 2010).

The precise mechanisms responsible for modality compatibility effects on dual-task performance are still under debate (Huestegge & Koch, 2010; Schacherer & Hazeltine, 2019; Stelzel & Schubert, 2011; Stephan & Koch, 2010). Based on ideomotor theory, current approaches focus on the role of crosstalk between stimuli and anticipated action effects between the component tasks (Schacherer & Hazeltine, 2020). According to this logic, an auditory-vocal task is modality compatible because the auditory effects of a vocal response overlap with the perception of the auditory stimulus modality, while the visuospatial effects of a manual response in an auditory-manual task are non-overlapping, thus forming a modality incompatible task. The cognitive demand to disentangle the overlap between sensory input modality, anticipated response effects and actual responses between two concurrently relevant task sets (Greenwald & Shulman, 1973; Hommel, 1998; Prinz, 1990) might be one central component of the involved control demands (Schacherer & Hazeltine, 2020). Our findings (Stelzel et al., 2017) indicate (i) that modality mappings also affect performance in continuous working-memory tasks, more similar in nature to maintaining postural stability than single-trial response-selection tasks and (ii) that task load and modality compatibility mappings both interfere with postural control in old age. Interference was highest, when cognitive task load was high (dual one-back task) and control demands were high (modality incompatible dual task). This suggests that the demands to coordinate multiple demanding task mappings in working memory with postural control contribute substantially to cognitive-postural interference in old age. Accordingly, researchers should design and develop cognitive-postural multitasking interventions involving specific modality pairings for older adults.

Single mode physical or cognitive training programs are effective in improving motor and/or cognitive performance in old adults (Ball et al., 2002; Granacher, Gruber, & Gollhofer, 2009). Specifically, physical training can attenuate age-related changes in postural control that result from declining visual, vestibular, and proprioceptive function (Shaffer & Harrison, 2007) or muscle weakness (Hurley, Rees, & Newham, 1998). Balance training, which typically involves exercises performed on stable and unstable surfaces, improves older adults’ balance performance, reduces deficits in postural control and lowers falls risk (DiStefano, Clark, & Padua, 2009; Granacher, Gruber, Strass, & Gollhofer, 2007; Hortobágyi et al., 2015; Lesinski, Hortobágyi, Muehlbauer, Gollhofer, & Granacher, 2015; Madureira et al., 2007; Steadman, Donaldson, & Kalra, 2003). To increase task difficulty during balance training, the base of support (e.g., bipedal, tandem, monopedal stance) and sensory input (e.g., eyes open vs eyes closed) are systematically manipulated (Granacher et al., 2007; Madureira et al., 2007; Muehlbauer, Besemer, Wehrle, Gollhofer, & Granacher, 2012). As evidence suggests that the effects of balance training in old adults are highly task-specific, balance exercises should mimic everyday tasks (Giboin, Gruber, & Kramer, 2015; Kümmel, Kramer, Giboin, & Gruber, 2016).

Standardized cognitive training interventions have successfully improved specific cognitive functions or general cognitive ability in old age (Ball et al., 2002; Kelly et al., 2014; Mozolic, Long, Morgan, Rawley-Payne, & Laurienti, 2011; Reijnders, van Heugten, & van Boxtel, 2013). However, training two cognitive tasks simultaneously is more effective in improving dual-task performance than training the same tasks separately in young (Jaeggi, Buschkuehl, Jonides, & Perrig, 2008; Liepelt, Strobach, Frensch, & Schubert, 2011; Schumacher et al., 2001) and older adults (Bherer et al., 2008; Strobach, Frensch, Mueller, & Schubert, 2012; Strobach & Schubert, 2017). Furthermore, training effects proved to be particularly robust if the training tasks were adapted to an individual level of performance (Holmes, Gathercole, & Dunning, 2009; Klingberg, 2010) and if interventions were task-specific (Rebok & Balcerak, 1989). Studies involving modality compatible and incompatible tasks revealed steep learning curves for both modality pairings, albeit showing that modality incompatible dual-task costs are more persistent over time (Göthe et al., 2016; Hazeltine et al., 2006). This emphasizes the high cognitive demands that are needed to coordinate modality incompatible mappings.

More recent evidence suggests larger transfer from combined cognitive-postural training interventions to multi-task situations compared to single-mode interventions (Bherer, 2015; Granacher, Muehlbauer, et al., 2010). Additionally, larger transfer effects have been reported to occur between domains (Brauer & Morris, 2010; Li et al., 2010; Suo et al., 2016). However, to date no study has compared cognitive-postural training interventions that vary in regards of compatibility between stimulus input and motor output. Therefore, the goal of this study was to explore the effects of two cognitive-postural training interventions with varying multitasking conditions in healthy older adults. More specifically, we investigated the effects of multi-task balance training on the ability to coordinate multiple task mappings under increased postural demands in older adults.

For this purpose, healthy older adults aged 65-85 years were either assigned to a modality compatible or a modality incompatible training group. After baseline testing (T0), all participants first underwent a passive control period for six weeks before they were retested (T1). Subsequently, participants performed modality compatible or incompatible training for six weeks. After having completed the training, participants were tested a third time (T2). With reference to the relevant literature (Granacher, Muehlbauer, et al., 2010; Strobach et al., 2012), we expected that older adults would improve cognitive performance, balance, and reduce associated dual-task costs during the intervention period (T1 vs. T2) compared to the passive control period (T0 vs. T1). Based on previous research (Hazeltine et al., 2006), it was hypothesized that the largest training effects would be observed for conditions that displayed congruency between task compatibility and training group, i.e. the modality incompatible training group would improve to a greater extend in situations involving modality incompatible dual tasks than the modality compatible training group and vice versa.

## Materials and Methods

### Participants

Older adults were recruited via newspaper advertisements in Potsdam and Berlin, Germany for a large-scale study involving electroencephalographic (EEG) and functional magnetic resonance imaging (fMRI) measurements (Bohle et al., 2019; Stelzel et al., 2018). Study eligibility was examined with a standardized protocol. Participants were included if no relevant diseases (e.g., neurophysiological, psychiatric, cardiovascular, vestibular/gait disorders) were reported and no psychopharmacological medication was taken. Participants were excluded if they had no normal hearing abilities, no normal or no corrected-to-normal vision or if they had a score of < 27 in the Mini-Mental State Examination Test (Folstein, Folstein, & McHugh, 1975). Eligibility for the fMRI study was determined separately based on the rules and safety guidelines of the Berlin Center for Advanced Neuroimaging (BCAN).

We conducted an a priori power analysis with an assumed Type I error of .05, a Type II error rate of .20, and an intra-subject correlation coefficient of .7 for the changes from baseline observed at the post-intervention assessment for balance measurements (Granacher, Muehlbauer, et al., 2010). Adjusting for a potential dropout rate of 10%, we calculated that 20 persons per group would be sufficient for finding a statistically significant treatment effect with a probability of 80% provided the average treatment effect at the post-intervention assessment equals one standard deviation of the random subject effect.

The recruitment of suitable individuals proved to be difficult due to strict cognitive and physical exclusion criteria as well as the length of the experiment. From 41 participants who initially volunteered to participate in the experiment, 17 individuals withdrew or had to be excluded prior to the start of the study. Twenty-eight older adults (14 female, 14 male) aged 65 to 85 years (mean [M]: 72.0 ± 5.5 years) met the inclusion criteria and started the training intervention. Participants were assigned to one of two training groups (modality compatible, modality incompatible) using a stratified pseudorandom sampling strategy controlling for gender and age. One participant (f) withdrew after two sessions because of health issues unrelated to the intervention. Four other participants (f = 1, m = 3) withdrew from the study for personal reasons (e.g., not sufficient time). Two participants completed the training intervention but were excluded from the final sample because their data met the previously defined exclusion criteria (see Data and Statistical Analysis). Therefore, the final sample consisted of 21 old adults. The study was designed according to the latest version of Declaration of Helsinki and approved by the University of Potsdam ethics committee (Approval Number 20/2015). All participants were informed and provided their written consent. Study participation was financially awarded with a total amount of 120 €.

Notably, sixteen participants of the final cohort also participated in the fMRI experiment within three weeks after T0. Here, participants performed cognitive single and dual tasks with compatible and incompatible modality mappings that were identical to those performed during the test sessions at T0, T1 and T2. Thus, these individuals had additional time to practice cognitive tasks compared to those, who only participated in the intervention study (n = 5). Group-specific demographic, neuropsychological and physical performance data are presented in ***Table 1***.

**Table 1:** Demographic, neuropsychological, and physical performance data of the two training groups at T0.


TRAINING GROUP	MODALITY COMPATIBLE (N=11)	MODALITY INCOMPATIBLE N = 10	*P*-VALUE

**Age (years)**	71.1 (±6.2) range = 65–83 yrs	73.1 (±4.8) range = 66–79 yrs	.42

**Sex**	female = 4 (36.3%) male = 7 (63.6%)	female = 6 (60%)male = 4 (40%)	.30

**Neuropsychology**^1^			

Trail making test A (s)	37.7 (8.6)	46.1 (15.1)	.13

Trail making test B (s)	75.2 (22.5)	109.0 (35.5)	**.02**

CERAD immediate recall (# words)	7.8 (1.0)	8.0 (1.1)	.69

CERAD delayed retrieval (# words)	7.6 (2.0)	8.00 (2.4)	.64

DSST (# correct matches in 90s)	49.3 (5.9)	39.8 (9.0)	**.01**

LPS subtest 3 (# correct symbols)	20.6 (4.3)	21.9 (5.5)	.54

MWT (# correct words)	32.7 (1.5)	32.9 (2.1)	.83

DST forward (# of digits)	7.6 (1.7)	8.6 (2.5)	.30

DST backward (# of digits)	6.2 (1.2)	6.6 (2.3)	.60

MMSE (points)	28.9 (1.3)	28.7 (1.1)	.69

**Physical Performance**			

10 m walk test [DT(s)-ST(s)]	12.6 (17.9)	8.3 (10.0)	.51

Timed Up and Go Test (s)	6.6 (1.1)	7.2 (1.3)	.31

Hand grip strength test (kg)	30.4 (9.7)	32.2 (10.9)	.69


^1^ ANCOVAs with PCA factor scores representing neuropsychological status at baseline (see Table 4) as covariates did not reveal any significant effects for training group on the outcomes of the training intervention.

### Design and Procedure

All test and training sessions were conducted at the biomechanics laboratory, University of Potsdam, Germany. The study consisted of three test sessions (T0, T1, T2), with T0 being split in two test days separated by 7 to 28 days. On day 1, participants completed a battery of neuropsychological tests, i.e. immediate recall (three trials) and delayed retrieval of the German version of the ‘Consortium to Establish a Registry for Alzheimer’s Disease’ (CERAD) word list (Morris, Mohs, & Rogers, 1989), Trail Making Test (part A & B) (Reitan, 1958), Stop-Signal Reaction-Time (SSRT) Task (Logan & Cowan, 1984), the Figural Relations Subtest of the German Intelligence Test (*Leistungsprüfsystem*, LPS) (Horn, 1983), a Multiple Choice Vocabulary Test (*Mehrfachwahl-Wortschatz-Intelligenztest*, MWT) (Lehrl, 1999), as well as the Digit Span Test (DST, forward and backward) and the Digit Symbol Substitution Test (DSST) from the Wechsler Adult Intelligence Scale (Wechlser, 1955). Motor tests included assessing 10-m walking speed under single- and dual-task conditions involving serial subtractions by three (Beurskens, Steinberg, Antoniewicz, Wolff, & Granacher, 2016), the Timed Up & Go (TUG) Test (Podsiadlo & Richardson, 1991), and the assessment of hand grip strength using a dynamometer. Additionally, general hearing ability, vision, and general cognitive functioning in the Mini Mental State Examination (MMSE) was assessed (Folstein et al., 1975). At the end of the first test day, participants practiced the experimental cognitive tasks applied in the test sessions (see below). On day 2 of session 1, leg dominance was identified using Coren’s lateral preference inventory (Coren, 1993). Subsequently, participants performed the experimental tasks while recording electroencephalographic (EEG) and center of pressure (CoP) data (for EEG at T0, see (Bohle et al., 2019)). After the passive control period participants were tested again using the same experimental paradigm (test session T1). During the passive control period, participants were asked to not change their lifestyle behavior (e.g., physical activity). After the intervention, participants completed the experimental paradigm one last time (test session T2). The study design is illustrated in ***Figure 1a***.

**Figure 1a:**
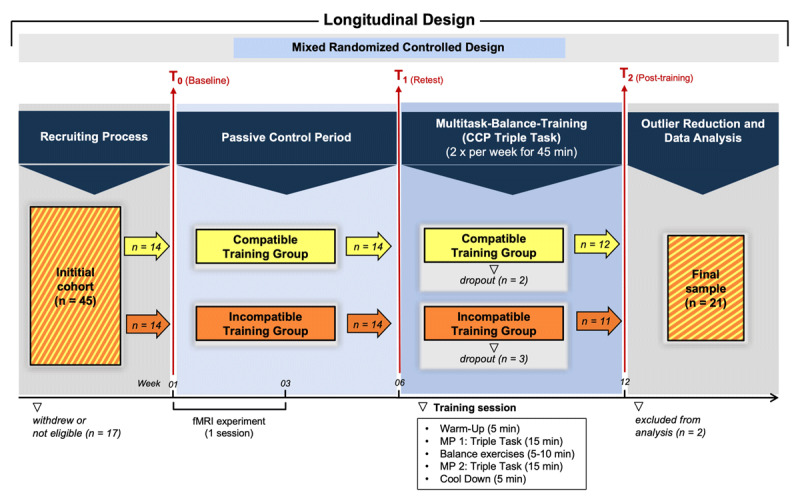
Study design. After being recruited, participants were assigned to a compatible or incompatible training group. They were assessed for cognitive and postural performance and neuropsychological status at baseline (T0) and subsequently underwent a 6-week passive control period. After retesting (T1), they completed a 6- week multimodal balance training intervention and were tested a third time (T2).

### Experimental Paradigm

Presentation software (*https://www.neurobs.com*) was used for presenting visual and auditory tasks and for recording manual and vocal responses. Participants wore headphones with an attached microphone to record vocal responses trial-wise and were equipped with a response key in their right hand, which allowed them to press a button with their right thumb. Correct and false responses as well as reaction times (RTs) from vocal response data were analyzed by a self-developed and validated Matlab tool (Reisner & Hinrichs, 2016). Given that participants always held a response key during the testing of postural sway, a potential effect of pressing the key on CoP data can be ruled out.

The experimental design (see ***Figure 1c***) was identical across all test sessions and consisted of a within-subject block design, including two parts (one with modality compatible tasks, one with modality incompatible tasks, see below). The order of the two parts was counterbalanced between participants. Each part consisted of six runs – three runs in standing posture and three runs in sitting posture. All participants started in standing posture in each part and posture was alternated after each run. In standing posture, two single postural task blocks were presented at the beginning and the end of each run, respectively. In between, participants performed two blocks of cognitive-postural dual tasks and one block of the cognitive-cognitive-postural triple task (see below), including the respective modality compatible or incompatible input-output modality mappings for 16 trials. This resulted in seven task blocks for standing posture runs with a duration of 33 seconds per block. In sitting posture, participants only performed the respective cognitive single tasks or the cognitive-cognitive dual task. The latter three block types were counterbalanced in their order between runs. For example, the cognitive-cognitive dual task with modality compatible input output pairings was presented once at the first, second and third position of the modality compatible sitting and standing posture runs, respectively.

The order of task blocks and the trial order within each block were kept the same across participants. Both parts lasted 30-40 min each and were separated by a break of several minutes.

#### Postural Single Task (P)

Participants stood in semi-tandem stance on a balance pad with the dominant leg placed posterior and arms hanging loosely beside the body. Participants were instructed to keep their head straight and fixate a stable (fixation cross) or dynamic (alternating fixation cross and ampersand symbol) visual stimulus for 33 seconds each. The presentation times in the dynamic condition were identical to the one-back task (1500 ms fixation cross, 500 ms ampersand symbol). Only data of the dynamic stimulus condition will be reported as postural baseline task, see ***Figure 1b***.

**Figure 1b:**
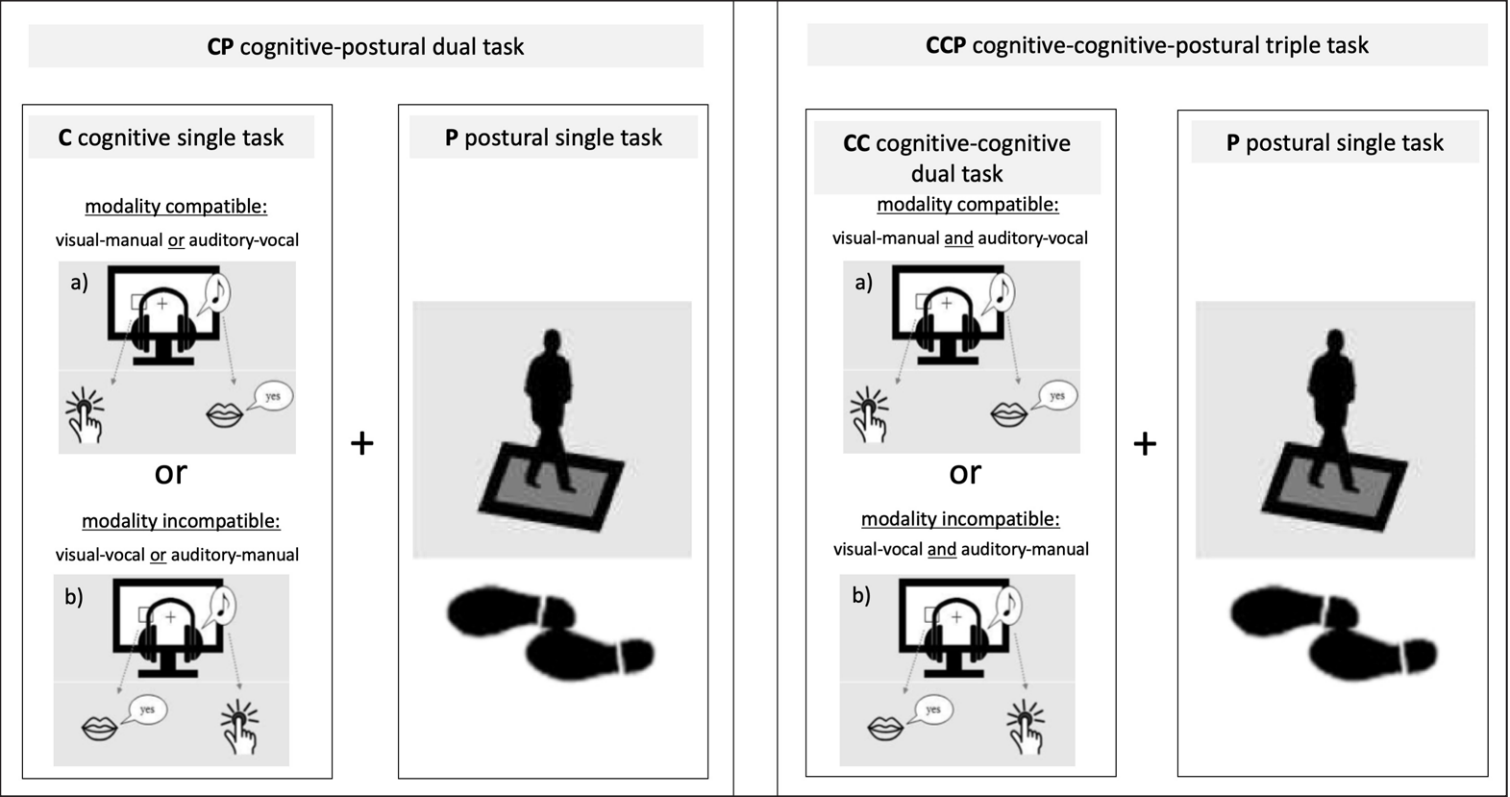
Task design. Variations of modality compatible and modality incompatible component one-back working memory tasks either performed as cognitive single task (C), cognitive-postural dual task (CP), cognitive-cognitive dual task (CC) or cognitive-cognitive-postural triple task (CCP) at T0, T1, and T2. Input stimuli were either visual or auditory with either manual or vocal (‘yes’) response output requirements. Visual displays consisted of 6 possible stimulus locations, 3 to the left and 3 to the right of the fixation cross presented for 500 ms, followed by a 1,500 ms inter-stimulus interval. Auditory input stimuli consisted of three different tones (200, 450, 900 Hz), presented via headphones to the left or to the right ear. Participants were asked to respond as fast and correct as possible to one-back targets via button press in the manual conditions or by saying “yes” in the vocal conditions. Depending on the part of the experiment, stimulus-response mappings were either modality compatible (i.e., visual-manual and auditory-vocal) or modality incompatible (i.e., visual-vocal and auditory-manual). One block lasted 33s and included 16 stimuli with five target stimuli.

**Figure 1c:**
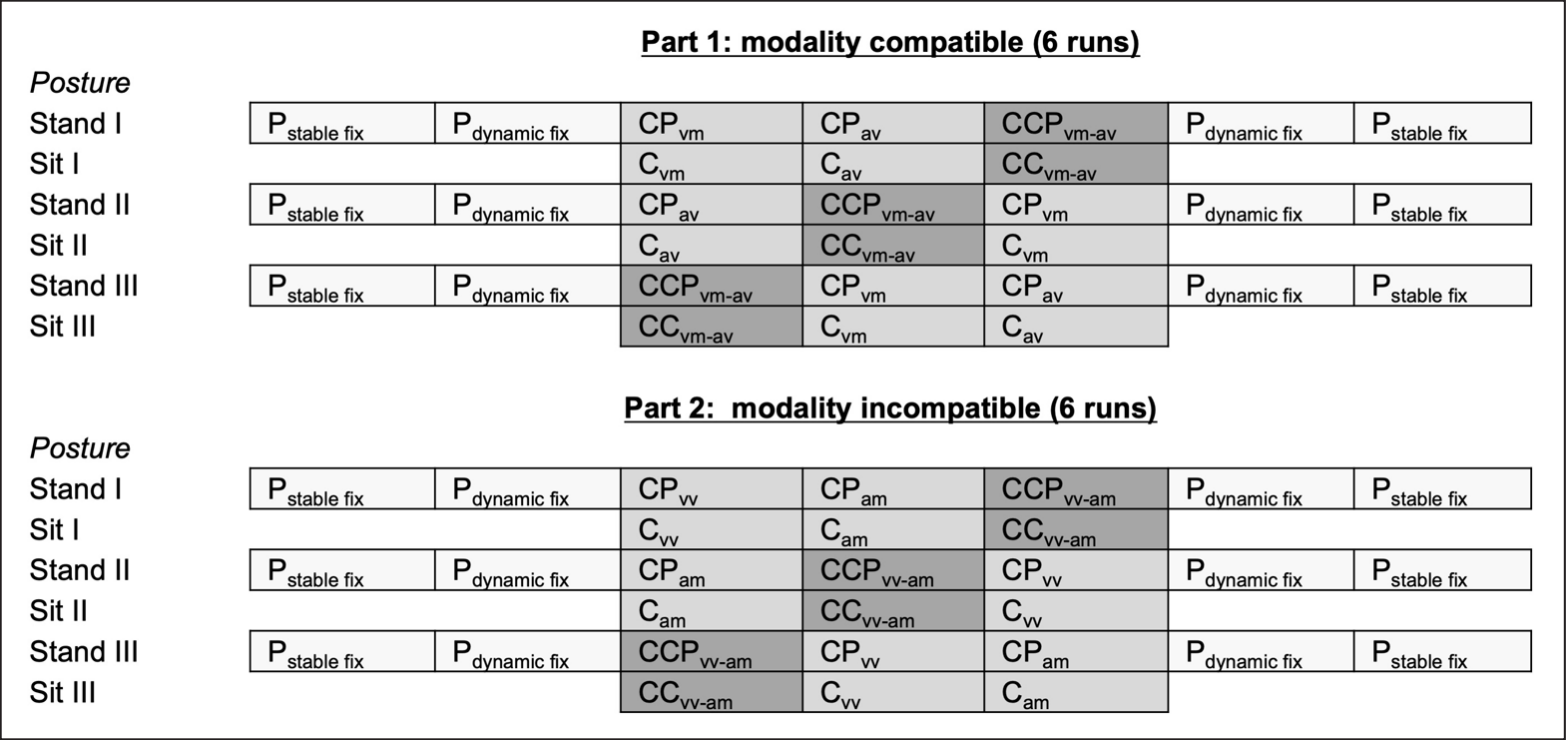
Design of test sessions (T0, T1, T2). Each test session included two parts (modality compatible, modality incompatible). Each part consisted of six runs (three in standing posture, three in sitting posture). Task blocks (33 secs) within each run were processed from left to right. Each run in standing posture included seven task blocks and each run in sitting posture three task blocks each. Each task block consisted of 16 trials (i.e. stimulus presentation). Explanations for Abbreviations are as follows: P_stable fix_ = postural single task with stable fixation, P_dynamic fix_ = postural single task with dynamic fixation; CP_vm_ = cognitive-postural dual task with visual-manual stimulus-response pairing of cognitive task, CP_av_ = cognitive-postural dual task with auditory-vocal stimulus-response pairing of cognitive task, CCP_vm-av_ = cognitive-cognitive-postural triple task, CP_vv_ = cognitive-postural dual task with visual-vocal stimulus-response pairing of cognitive task, CP_am_ = cognitive-postural dual task with auditory-manual stimulus-response pairing, CCP_vv-am_ = cognitive-cognitive-postural triple task. For further descriptions of tasks see Methods section.

#### Cognitive Single Task (C)

Participants performed visual or auditory one-back working memory tasks while sitting on a chair with a backrest. The input stimuli were visual or auditory with either manual or vocal (‘yes’) response output requirements. Visual stimuli (white squares on a black screen, presentation times: 500 ms) were displayed in one of six positions (left or right side; bottom, middle, up). Auditory input stimuli consisted of three different tones (200, 450, 900 Hz), presented via headphones to the left or to the right ear. Stimulus-response mappings were either modality compatible (i.e., visual-manual and auditory-vocal) or modality incompatible (i.e., visual-vocal and auditory-manual), see ***Figure 1b***. Participants were asked to respond as fast and correct as possible. Each block of 16 trials contained five one-back targets.

#### Cognitive-Postural Dual Task (CP)

Participants had to process either the visual or the auditory single task (C), while simultaneously performing the postural single task (P), see ***Figure 1b***.

#### Cognitive-Cognitive Dual Task (CC)

While sitting on a chair, participants were instructed to perform the visual and auditory cognitive task (C) simultaneously for 500 ms, followed by a 1,500 ms inter-stimulus interval. Each block contained two/three target stimuli in the visual modality and two/three in the auditory modality, pseudo-randomized across blocks. Visual and auditory target-stimuli were never presented simultaneously. Stimulus-response mappings were either modality compatible (i.e., visual-manual and auditory-vocal) or modality incompatible (i.e., visual-vocal and auditory-manual), see ***Figure 1b***.

#### Cognitive-Cognitive-Postural Triple Task (CCP)

In the CCP task condition, participants were asked to perform the CC task and the P task simultaneously (***Figure 1b***).

### Training Intervention

**Figure 2:**
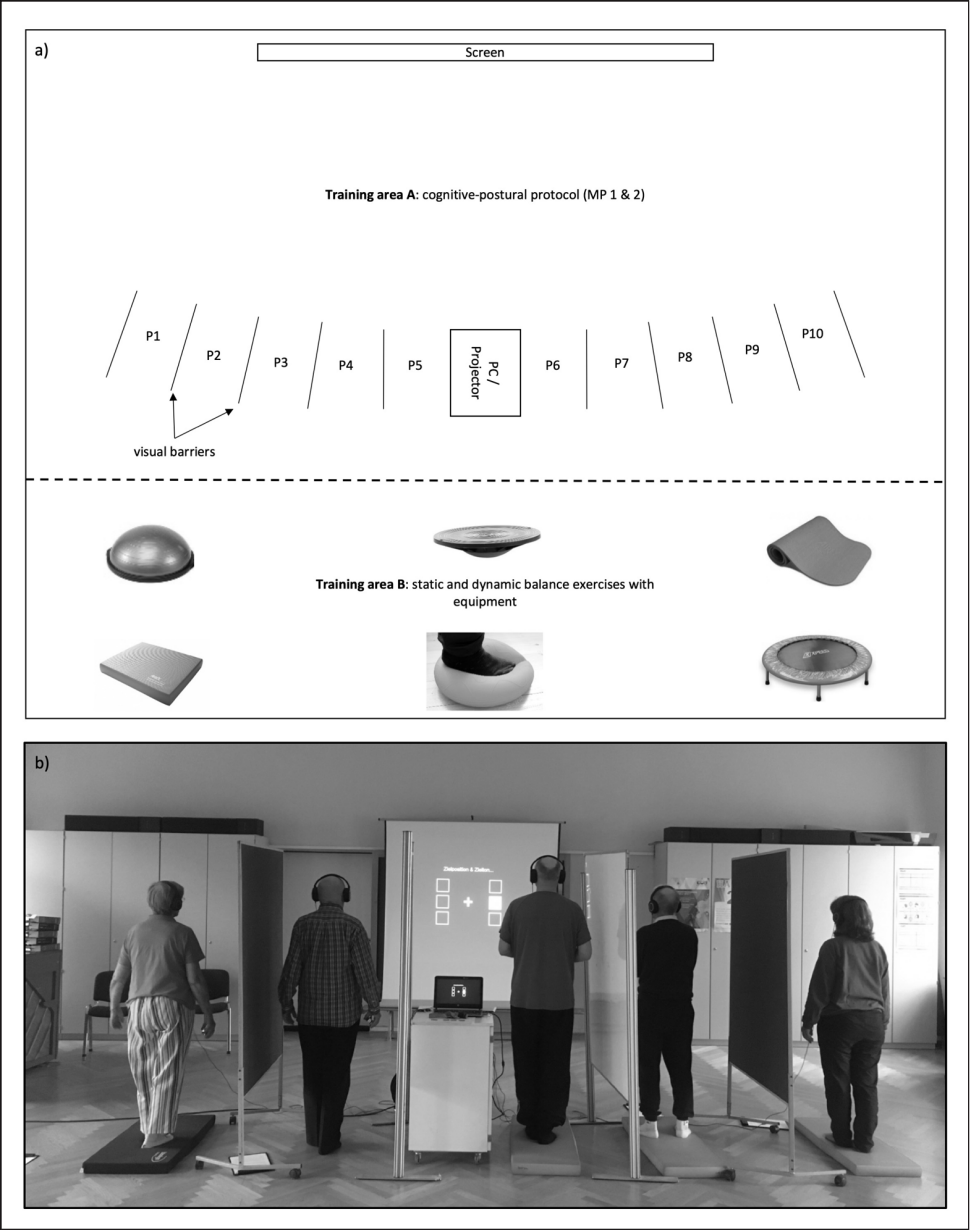
Setup during a training session. Separated by visual barriers, participants (P1–P10) stood next to each other in the exercise room to perform the two main parts of the training session. Between main part 1 and main part 2, participants completed a circuit consisting of static and dynamic balance exercises in training area B using various equipment. Participants provided written consent for the publishing of the picture.

Participants completed a progressive combined balance-cognition training program including modality compatible or modality incompatible CCP tasks. The program duration was six weeks, with a total of 18 training sessions and three sessions a week. The program took place in a gym at the University of Potsdam and was supervised by trained professionals (i.e., sport scientists with a Master’s degree). Each session lasted approximately 50 min and was attended by a maximum of 10 participants. After a short general warm up (5 min), participants completed the first main part (MP 1), which lasted about 15 minutes. Separated by partition walls, participants stood next to each other and faced a projection screen set up at a distance of 3 m (***Figure 2***). Participants were equipped with noise canceling headphones and held a button in their right hand, with which they responded to the presented visual or auditory stimuli when manual responses were required (visual-manual or auditory-manual tasks, respectively). Subsequently, participants performed different static and dynamic balance exercises for 5–10 minutes. This included walking through an obstacle course using unstable equipment such as balance pads, sissles etc. These exercises were performed at a low intensity to prevent fatigue. Afterwards, the second main part (MP 2) of the training paradigm was completed, which was identical in structure to MP 1. At the end of the training session, participants completed a 5-minute cool down, which consisted of dynamic stretching exercises.

The training intervention consisted of ten different training levels (L), see ***Table 2***. Each training level involved a specific combination of cognitive and postural tasks. Cognitive tasks included a 0-, 1-, or 2-back working memory dual task, identical to the experimental paradigm (CC) described above. Note that the same set of stimuli within one task block was used for the performance of the different n-back task types, as training levels could differ between individuals within one session. The modality compatible training group only trained the modality compatible dual tasks while the modality incompatible training group only trained the modality incompatible dual tasks. During each part of the training session, participants performed 12 dual-task blocks with 16 trials each. Postural tasks included the bipedal, the semi-tandem and the tandem stance as well as the one-legged-stance performed either on the level floor or on a balance pad. Each participant started their training on the easiest level ‘L1’. After each main part, rate of perceived exertion (RPE) was measured on a 10-point Likert scale. If participants rated their effort as ≤5, the training level was increased in the following main part. If the perceived effort was ≥8, the training level was decreased in the next main part. Due to technical constraints, no detailed cognitive or postural performance data were assessed in these group sessions.

**Table 2:** Adaptive Training Paradigm.


TRAINING LEVEL	COGNITIVE TASK	BALANCE TASK

L1	0-back Dual Task	bipedal parallel stance

L2	0-back Dual Task	bipedal parallel stance on balance pad

L3	1-back Dual Task	bipedal parallel stance on balance pad

L4	1-back Dual Task	semi-tandem stance

L5	1-back Dual Task	semi-tandem stance + balance pad

L6	2-back Dual Task	semi-tandem stance + balance pad

L7	2-back Dual Task	tandem stance

L8	2-back Dual Task	tandem stance + balance pad

L9	2-back Dual Task	one-legged-stance

L10	2-back Dual Task	one-legged-stance + balance pad


### Data Acquisition and Statistical Analyses

A one-dimensional force plate (Leonardo 105 Mechanograph®; Novotec Medical GmbH, Pforzheim, Germany) was used to measure postural sway, i.e., total center of pressure (CoP displacements), during bipedal stance at a sampling rate of 800 Hz. A balance pad (Airex®) was placed on the force plate to increase task difficulty. Total CoP displacements (mm) were computed using data for medio-lateral and anterior-posterior directions. A test duration of 33 s was chosen to comply with the cognitive task requirements and to achieve acceptable reliability of postural stability measurements (LeClair & Riach, 1996). If participants lost their balance, the test block was excluded from further data analyses.

Cognitive performance data during the test sessions (T0, T1, T2) were calculated as p(hit) – p(False Alarm(fa)) in the one-back target detection task. In addition to these performance measures, mean RTs for correct target responses are reported. Data were averaged for both component tasks of each modality compatibility condition, resulting in four performance measures for the modality compatible and modality incompatible condition, respectively (C, CP, CC, CCP). Cognitive performance reflects performance in target and non-target trials likewise.

Dual-task costs for CCP in the cognitive domain were calculated as relative performance (Perf) decrements in dual compared to single tasks (in %) according to the formula DTC_CCP_ = ([Perf_C_ – Perf_CCP_]/Perf_C_) ∗ 100. Triple-task costs of total CoP displacements, representing the difference between CCP and P, were calculated as TTC_CCP_ = ([CoP_CCP_ – CoP_P_]/CoP_P_) ∗ 100. Additionally, training-specific changes (training gain) in dual- and triple-task costs were calculated using the following equations: DTC_gain_ = (DTC_T1_ – DTC_T2_) – (DTC_T0_ – DTC_T1_) and TTC _gain_ = (TTC_T1_ – TTC_T2_) – (TTC_T0_ – TTC_T1_). These variables were then z-standardized and entered into separate repeated measures ANOVAs.

Prior outlier correction was as follows: Cognitive performance data were excluded block-wise if p(hit) < .3 and p(fa) > .3. Additionally, blocks were excluded if the recorded vocal responses were inaudible or when trials were interrupted due to technological errors. Postural data were excluded from the analysis if the recorded CoP displacements were ≥2 SDs of the grand mean. Further, blocks during which participants lost balance or touched an external object, such as the chair or the nearby wall, were excluded. A total of sixteen blocks of cognitive performance data (T1 = 11; T1 = 4; T2 = 1) and four blocks with CoP data at T0 were excluded.

As our research question addressed multi-task performance under increased postural demands, the main analysis focused on data collected in the CCP condition. To address whether any robust training effects were present, we first adjusted our data for the effects of modality compatibility by calculating the average performance of compatible and incompatible tasks This data was then entered in our main analysis, which consisted of a 3 (Time [T0, T1, T2]) × 2 (Training group [compatible training, incompatible training]) mixed ANOVA. We subsequently explored the effects during the passive control period (T0 vs. T1) and the training period (T1 vs. T2) separately, using 2 (Time [T0, T1 or T1, T2, respectively]) × 2 Modality mapping [compatible mapping, incompatible mapping] × 2 Task [ST, DT] × 2 Posture [sit, stance] × 2 Training group [compatible training, incompatible training]) mixed ANOVAs. Cognitive performance, calculated as p(hit)–p(fa), reflects performance in target and non-target trials likewise. The significance level was set at p < .05.

In an effort to explore the association between participants’ neuropsychological status and the outcomes of the training intervention, we reduced the dataset by entering six neuropsychological tests (LPS, TMT-A, Trail TMT-B, DSST, DTA-A, DST-B) assessed at baseline (***Table 1***) into a principal component analysis (PCA) with Kaiser-normalized Varimax rotation. Follow-up analyses were used to identify significant correlations between the obtained factors and dependent variables reflecting training gain.

## Results

In the following, findings from the main 3 (Time [T0, T1, T2]) × 2 (Training group [compatible training, incompatible training]) mixed ANOVA are summarized first. Afterwards, the results of our follow-up analyses are presented separately for the passive control (T0 vs. T1) and training periods (T1 vs. T2) including all task conditions. For the main analysis, all dependent variables were transformed to represent the average performance of incompatible and compatible tasks. For exploratory analyses, cognitive performance measures include absolute performance measures (p(hit)-p(fa)) as well as dual-task costs as primary outcomes and RTs as secondary outcome. For postural performance, mean (± SD) values for CoP path lengths and associated costs are reported. ***Table 3*** provides an overview of the statistical results for the main and exploratory follow-up analyses. Finally, associations between neuropsychological baseline measures and interindividual differences in training gains are reported.

**Table 3:** Significant main and interaction effects for cognitive and postural performance data.


FACTOR/INTERACTION	F-VALUE	P	ηP^2^

**Main Analysis 3 (Time) × 2 (Training Group) ANOVA (CCP condition only)**			

*Cognitive Performance*			

Time	F(1,19) = 16.70	<.001	.47

*Postural Performance*			

Time	*F*(1,19) = 3.79	.031	.17

*DTC (Costs)*			

Time	*F*(1,19) = 4.24	.022	.19

**Exploratory Follow-Up Analysis of Control Period (T0 vs. T1) (all task conditions)** **2 (Time) × 2 (Modality Compatibility) × 2 (Task) × 2 (Posture) × 2 (Group) ANOVA**			

*Cognitive Performance*			

Time	*F*(1,19) = 13.46	.002	.42

Task (ST vs. DT)	*F*(1,19) = 72.05	<.001	.79

Compatibility	*F*(1,19) = 7.96	.011	.30

Posture	*F*(1,19) = 38.34	<.001	.67

Time × Task	*F*(1,19) = 22.63	<.001	.54

Compatibility × Task	*F*(1,19) = 17.28	.001	.476

Posture x Task	*F*(1,19) = 32.77	<.001	.63

*DTC (Costs)*			

Time	*F*(1,19) = 22.51	<.001	.54

Compatibility	*F*(1,19) = 15.33	.001	.45

*Reaction Times*			

Task	*F*(1,19) = 324.78	<.001	.95

Task x Compatibility	*F*(1,19) = 12.73	.003	.44

*Postural Performance*			

Task	*F*(1,19) = 21.42	<.001	.53

**Exploratory Follow-Up Analysis of Training Period (T1 vs. T2) (all task conditions)** **2 (Time) × 2 (Modality Compatibility) × 2 (Task) × 2 (Posture) × 2 (Training Group) ANOVA**			

*Cognitive Performance*			

Task	*F*(1,19) = 65.41	<.001	.76

Posture	*F*(1,19) = 17.18	.001	.48

Posture × Task	*F*(1,19) = 7.35	.014	.28

*DTC (Costs)*			

Compatibility	*F*(1,19) = 17.23	.001	.48

*Reaction Times*			

Task	*F*(1,19) = 596.00	<.001	.97

Task x Compatibility	*F*(1,19) = 14.53	.001	.46

*Postural Performance*			

Task	*F*(1,19) = 16.95	<.001	.47


### Main 3 (Time) × 2 (Training Group) ANOVA

Cognitive performance in semitandem stance (CCP) changed significantly over time, *F*(1,19) = 16.70, *p* < .001, {{\eta}_p^2} = .47. Follow up t-tests revealed that cognitive performance increased significantly from T0 (M = .53; SD = .20) to T1 (M = .69; SD = .19), *t*(21) = –5.57, *p* < .001, *d* = 0.82, as well as from T0 to T2 (M = .68; SD = .16), *t*(21) = –4.54, *p* < .001, *d* = 0.83, but not from T1 to T2. Likewise, DTCs in the cognitive domain changed significantly over time, *F*(1,19) = 4.24, *p* = .022, {{\eta}_p^2} = .19, with T0 (M = 42.19; SD = 18.10) differing significantly from T1 (M = 28.93; SD = 17.39), *t*(21) = 4.86, *p* < .001, *d* = 0.74, but no difference with respect to T2 (M = 30.11; SD = 14.82). Regarding posture, there was a significant main effect for time on CoP path length exhibited in the CCP condition, *F*(1,19) = 3.79, *p* = .031, {{\eta}_p^2} = .17. CoP path length increased from T0 (M = 914.86; SD = 293.90) to T1 (M = 958.70; SD = 351.81) and T2 (M = 995.60; SD = 343.00), but only the difference between T0 and T2 reached significance *t*(21) = –2.849, *p* = .010, *d* = 0.25, respectively.

No significant main effects or interactions were found for triple-task costs associated with postural control or reaction time. There were no Group by Time interactions or any main effects for Group on any of the analyzed variables, which suggests that both training groups behaved similarly during the experiment. The significant results of the main analysis are summarized in ***Table 3***. ***Figure 4*** shows the group means and individual trajectories of postural sway and cognitive performance in the CCP condition for both the control and training period.

### Control Period (T0 vs. T1)

#### Cognitive Performance

Overall, findings from the exploratory analysis suggest that cognitive performance was significantly better at T1 (M = .85; SD = .11) compared to T0 (M = .76; SD = .15), for cognitive single tasks (M = .95; SD = .11) compared to cognitive dual tasks (M = .67; SD = .24), for modality compatible tasks (M = .83; SD = .21) compared to modality incompatible tasks (M = .78; SD = .25) and for sitting (M = .84; SD = .21) compared to standing (M = .77; SD = .26), see ***Table 3***.

The difference between dual-task and single-task performance was lower at T1 (mean difference between single and dual tasks: M = .22; SD = .14) compared to T0 (M = .34, SD = .17). However, the follow-up analysis revealed that this effect was independent of the modality mapping condition, indicating a general performance benefit for cognitive dual tasks after the passive control period. Likewise, dual-task costs were smaller after the passive control period (M = 28.95; SD = 19.98) compared to baseline (M = 43.57; SD = 23.02).

As expected, effects of modality mapping on cognitive performance were only present in the cognitive dual-task condition (difference between cognitive dual-tasks: M = .12, SD = –.03) but not in the single-task condition (M = –.01, SD = .27). Follow-up tests revealed a significant difference in compatibility effects between cognitive single and dual tasks, *t*(21) = 4.20, *p* < .001, *d* = 2.45, confirming increased interference associated with compatibility of modality mappings in dual-task situations. Participants generally exerted higher dual-task costs in incompatible (M = 44.52 SD = 23.59) compared to compatible modality mappings (M = 27.99; SD = 19.30). However, this effect did not interact with factor Time, showing a comparable reduction in dual-task costs for the modality compatible and incompatible stimulus-response mappings.

The exploratory follow-up analysis suggests that the factor posture (sitting vs. standing) modulated the effects of task on cognitive performance. In particular, cognitive dual-task effects were more pronounced during standing (difference between single and dual-task: M = .33, SD = .15) compared to sitting (M = .23, SD = .15, comparison of dual-task effects between sitting and standing, *t*(21) = –5.68, *p* < .001, *d* = 0.68). Lastly, changes in cognitive performance between T0 and T1 were similar across groups. Also, the training groups did not differ with respect to dual-task costs exerted in the cognitive domain.

Participants demonstrated faster RTs in single- (M = 665.19; SD = 91.26) compared to dual-task (M = 931.25; SD = 84.77) conditions. The data also indicated more pronounced differences between ST and DT in incompatible compared to compatible modality mappings (***Table 3***). Mean RTs were similar at T0 (M = 795.57; SD = 75.26) and T1 (M = 798.01; SD = 89.39), for modality compatible tasks (M = 788.79; SD = 85.30) compared to modality incompatible mappings (M = 804.55; SD = 88.06) and for sitting (M = 797.84; SD = 91.14) compared to standing (M = 797.93; SD = 76.45). This indicates that there were no speed-accuracy trade-offs. RTs were not different between groups.

#### Postural Performance

Postural single tasks (P) produced shorter CoP path lengths (M = 828.21; SD = 287.39) compared to CP (M = 907.03; SD = 293.09) and CCP (M = 936.77; SD = 333.01). Post-hoc comparisons revealed that postural performance differed significantly between P and CP (M = –73.52; SE = 11.86), *p* < .001, as well as between P and CCP (M = –101.76; SE = 23.24), *p* = .001, but not between CP and CCP, *p* = .437. Triple-task costs associated with postural control did not reveal any significant main effects or interactions, which further indicates that postural performance was identical for T0 and T1, modality compatible and incompatible tasks as well as between training groups.

### Training Period (T1 vs. T2)

#### Training Intervention

Overall, adherence to the combined cognitive-balance training intervention was high in both training groups. On average, participants attended 97±5% of the 18 training sessions. Nine participants (90%) of the incompatible training and ten participants (91%) of the compatible training group attended more than 90% of the sessions (attendance range 83-100%). Fifteen participants completed all 18 sessions. Average attendance was not significantly different between groups.

Regarding the training progression from week 1 (average training levels of the first three training sessions) to week 6 (average training levels of the last three training sessions), there was a significant main effect for time, *F*(1,19) = 101.94, *p* < .001, {{\eta}_p^2} = .85. The main effect for Training group did not reach significance, indicating that participants progressed similarly across groups. On average, participants began training at level 2 (±1) in the first week and performed their last sessions at level 7 (±2). Mental and physical effort of the completed training sessions was rated as 5.50±1.21 (range = 2.83–8.44) and 5.10 ±.96 (range = 3.56–6.90), respectively. Average rest between sessions was 2.29 (± 2.03) days, which allowed for sufficient recovery. Repeated measures ANOVA with Time as within-subject factor and Training group as between-subject factor did not reveal any significant main effects, suggesting that participants exhibited a comparable physical and mental effort over the course of the training intervention, reflecting that the adaptive training protocol was implemented correctly.

#### Cognitive Performance

The exploratory analysis did not reveal any significant main effects for time, indicating that cognitive performance of participants was similar between T1 and T2. Likewise, a follow-up analysis did not show any significant effect for Training group or – in contrast to the T0-T1 period – modality mappings on cognitive performance, which suggests that cognitive performance was neither influenced by the compatibility of modality mappings in the testing condition nor by the different cognitive training protocols. However, cognitive performance was higher for cognitive single tasks (M = .95; SD = .11) compared to cognitive dual tasks (M = .72; SD = .21), and for sitting (M = .87; SD = .18) compared to standing (M = .81; SD = .22). Furthermore, cognitive dual-task effects were more pronounced during standing (difference between single and dual task: M = .26; SD = .13) compared to sitting [M = .19; SD = .14, comparison of dual-task effects between sitting and standing *t*(21) = -2.70, *p* = .014, *d* = .49], which suggests that the effect of task load on cognitive performance was influenced by the postural demands (sitting vs standing) of the task.

Participants exerted higher dual-task costs (M = 35.56; SD =21.35) in incompatible compared to compatible tasks (M = 22.79; SD = 17.08). However, cognitive dual-task costs were similar before and after the training intervention and were not affected by the compatibility of input-output modality mappings during the intervention.

Participants demonstrated faster RTs in single (M = 666.45; SD = 99.34) compared to dual-task (M = 920.01; SD = 93.01) conditions, with effects of task load (ST vs. DT RT) being more pronounced in incompatible compared to compatible tasks. RTs were similar at T1 (M = 798.01; SD = 89.39) and T2 (M = 786.23; SD = 92.64), for modality compatible tasks (M = 785.40; SD = 85.99) compared to modality incompatible tasks (M = 799.27; SD = 93.78) and for sitting (M = 800.63; SD = 96.94) compared to standing (M = 786.02; SD = 78.95). Training groups did not differ with respect to RT.

#### Postural Performance

**Figure 3:**
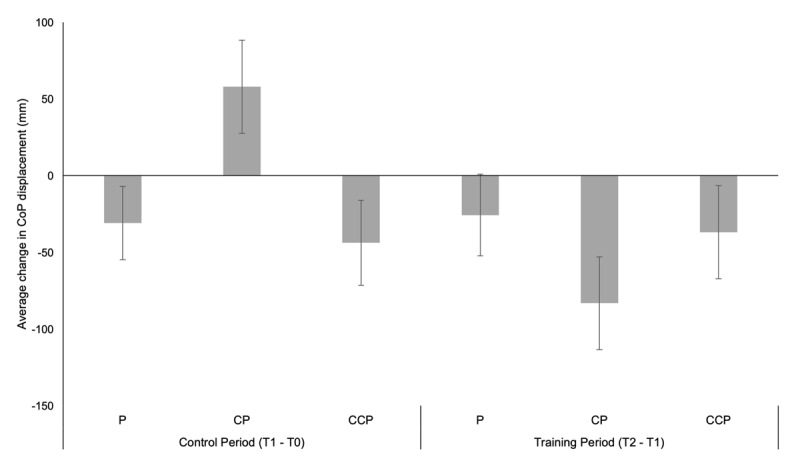
Mean (±SE) postural performance data. Changes in center of pressure (CoP) displacements for the control and training period.

Postural performance was lower in postural single tasks (P) (M = 856.60; SD = 290.94) compared to CP (M = 944.68; SD = 300.17) and CCP (M = 995.59; SD = 342.99). Exploratory analysis revealed that postural performance mainly differed between P and CP (M = –88.07; SE = 13.66), *p* < .001, as well as between P and CCP (M = –138.98; SE = 27.67), but not between CP and CCP (M = –50.90; SE = 19.50). An exploratory 2 (Time[T0,T1]) × 2 (Modality[compatible, incompatible]) × 2 (Group[compatible training, incompatible training]) ANOVA did not reveal any main effects for postural triple-task costs, which indicates that postural sway was similar before and after the training intervention, as well as for single and dual tasks. Furthermore, both training groups showed similar triple-task costs across task conditions and times of measurement. ***Figure 3*** compares the changes in CoP data for the training and control period for the different task conditions.

**Figure 4:**
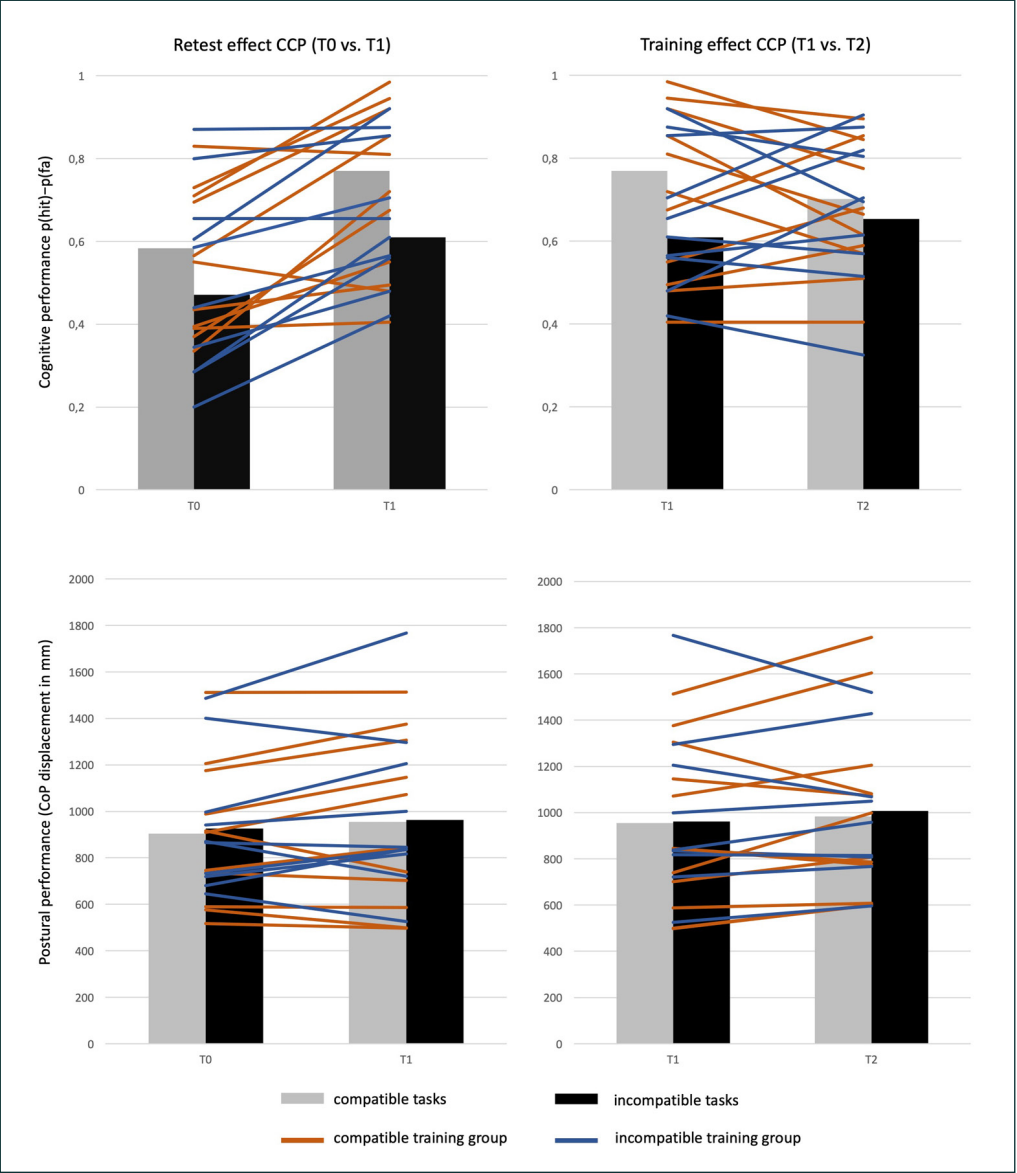
Individual trajectories of cognitive and postural performance in the cognitive-cognitive-postural triple task (CCP). Comparison of passive control period (retest effect) and training intervention (training effect).

### Neuropsychological Predictors of Training Responses

As shown in ***Figure 4***, interindividual variability in retest and training effects was substantial. To explore whether training-related effects were linked to participants’ cognitive status at T0, a principal component analysis (PCA) was conducted on six neuropsychological items (LPS, TMT-A, Trail TMT-B, DSST, DTA-A, DST-B) assessed at baseline. The Kaiser-Meyer-Olkin measure verified the sampling adequacy for the analysis, KMO = .77 (‘middling’ according to Hutcheson & Sofroniou, 1999), and all KMO values were greater than .681, which is well above the acceptable limit of .5 (Field, 2013). An initial analysis was run to obtain eigenvalues for each factor in the data. Two factors had eigenvalues over Kaiser’s criterion of 1 and in combination explained 60.75% of the variance. The scree plot showed inflexions that justified retaining three factors, and the overall variance explained with three factors reached 79.81%. ***Table 4*** shows the factor loadings after rotation. The items that cluster on the same factor suggest that factor 1 represents Working memory capacity, while factor 2 represents Attentional control and factor 3 represents Processing speed. Follow-up analysis indicates that Attentional control correlates moderately with gain in incompatible cognitive dual-task performance (r = .50, *p* = .024), indicating that individuals with high attentional control at baseline showed a greater training-specific improvement in the demanding modality incompatible dual task. Processing speed (r = .45, *p* = .046) and Working memory capacity (r = –S.47, *p* = .043) both correlated moderately with gain in balance under compatible task conditions. Note that the last factor is coded inversely, thus reflecting a converging finding of greater gain in balance for individuals with lower working memory capacity performance at T0. None of these correlations, however, withstands Bonferroni correction.

**Table 4:** Summary of PCA results for the neuropsychological tests conducted at baseline.


ITEM	ROTATED FACTOR LOADINGS			

	WORKING MEMORY	ATTENTIONAL CONTROL	PROCESSING SPEED

LPS (number of rows correctly identified)	**.82**	–.08	–.32

Trail Making Test A (time in s)	–.10	.29	.**93**

Trail Making Test B (time in s)	–.12	**.93**	.12

DSST (number of boxes filled in correctly in 90s)	.10	**–.89**	–.27

Digit Span A (score)	**.91**	.01	.10

Digit Span B (score)	**.87**	–.24	–.07

Eigenvalues	2.20	1.43	1.14

**% of variance**	36.98	23.78	19.06

α	.75	.57	.77


## Discussion

Although evidence suggests that cognitive-motor training compared to single-mode interventions offers greater benefits to older adults with respect to falls risk (Silsupadol et al., 2009), little is known about the effect of specific cognitive demands in this context. Here, we focused on the role of specific input-output modality pairings that have been shown to affect multitasking performance in the cognitive domain (Hazeltine et al., 2006; Stelzel et al., 2006; Stephan & Koch, 2010). Modality incompatible stimulus-response mappings interfere with each other to a greater extent than modality compatible mappings, and – in old adults – they also seem to interfere more with postural demands (Stelzel et al., 2017). Whether an adaptive cognitive-postural training intervention involving these demanding stimulus-response mappings leads to domain-specific effects is unknown but functionally and clinically highly relevant. To the best of our knowledge, this is the first study that investigated the effects of two separate intervention protocols, which employed modality compatible and modality incompatible cognitive-postural training tasks to address the research question, if and how these interventions affect cognitive and postural performance in older adults. We expected that systematic cognitive-postural training results in improved postural and cognitive performances as well as in reduced dual-task costs for both domains.

Our results do not support these specific hypotheses. Although our main analysis indicated changes in cognitive performance and associated DTCs over time, follow-up analyses of the control and training periods indicate that improvements occurred during the passive control period that preceded the training intervention. No further effects of the training intervention were present in the analysis of group means. Note however that the results of the exploratory follow-up analyses revealed cognitive performance patterns consistent with previous studies that investigated the effects of modality compatibility mappings (modality incompatible compared vs. modality compatible tasks), varying working memory load (cognitive single vs. cognitive dual task), and increased postural demands in old adults.

Finally, our analysis of individual differences in training gains revealed some promising results regarding training gains in a subset of individuals. Old adults with good performance in attentional control tasks, requiring the focused switching between visual inputs and working-memory contents, seem to improve to a greater extent in the cognitive performance of the demanding modality incompatible dual task. However, this trend was not associated with higher training gains in the postural domain. In contrast, old adults with higher ability in processing speed and those with lower working memory capacity seemed to benefit to a greater extend from the training intervention in the postural domain when concurrently performing less demanding modality compatible tasks. While these findings have to be interpreted with caution due to low statistical power, they could support the idea that individual cognitive status crucially affects the dynamic interplay in prioritization between the cognitive and the motor domains in complex cognitive-motor multitasking situations.

In the following, we will summarize and discuss our results with respect to the different research questions that were addressed in this experiment. First, we will focus on the general effects of the intervention by comparing the retest and training periods. Afterwards, we will separately discuss the effects of cognitive task load and postural demands, as well as those of modality compatibility mappings. Finally, we will focus on the large interindividual variability of training responses and establish recommendations for future research.

### Retest vs. Training Period

Our main analysis indicated a general main effect of time on measures of cognitive performance. However, follow-up analysis revealed the absence of a significant training effect (T2 – T1) in combination with a retest effect (T1 – T0) in the cognitive domain across training groups. This suggests that repeating the cognitive-postural task protocol after a passive control period of six weeks may affect cognitive performance of older adults, while mitigating potential training effects. Studies involving repeated neuropsychological testing have confirmed these so-called practice effects, which are defined as an increase in a participant’s performance between test administrations in the absence of any interventions (Bartels, Wegrzyn, Wiedl, Ackermann, & Ehrenreich, 2010). Notably, it has been shown that the strongest practice effects occur early during the repeated testing period and that these effects particularly affect executive functions, learning and memory (Bartels et al., 2010; Benedict & Zgaljardic, 1998), all of which played an important role in the current training intervention. In fact, previous studies have demonstrated that the most significant practice gains can be observed in the first testing session taking place 2-3 weeks after baseline testing. In contrast, practice gains in subsequent sessions are substantially smaller (Bartels et al., 2010; Benedict & Zgaljardic, 1998) which can be explained by the principle of diminishing returns. Although it has been argued that practice effects diminish with greater age (McCaffrey & Westervelt, 1995), evidence has shown that older adults exhibit significant improvements in common neuropsychological measures, including working memory tasks (Calamia, Markon, & Tranel, 2012; Duff, 2014), making it an important factor to consider when designing intervention studies involving older adults.

In this context, it is also important to mention that sixteen of the twenty-one participants in our cohort also participated in an fMRI experiment that was conducted during the passive control period (one to three weeks after T0). Given the research on practice effects, it appears reasonable that participation in the concurrent fMRI experiment may have contributed to the increase in cognitive performance between T0 and T1. Note however, that the applied tasks were not completely identical between test sessions. For example, the specific order of stimulus presentation and target presentation (i.e., the pseudo-randomization) as well as the context (biomechanics lab vs MRI) differed substantially between sessions. This raises the question whether this fast performance increase due to repeated task performance is functionally relevant and may transfer to situations with similar demands. However, this question cannot be answered by the present study design.

### Effects of Cognitive Task Load and Postural Demands

In an effort to improve our understanding of increased postural demands and task load, we performed follow-up analyses on cognitive data in seated and standing position during single- and dual-task situations. As expected, overall cognitive performance was lower in cognitive dual-task (CC, CCP) compared to cognitive single-task conditions (C, CP), when averaged across compatible and incompatible trials. Notably, this observation was persistent across all test occasions, that is, cognitive dual-task costs were not eliminated after training. This finding is consistent with previous dual-task training studies, which showed robust improvements in dual-task performance (Hazeltine et al., 2006; Liepelt et al., 2011; Strobach et al., 2012) but only very rarely ‘perfect-time sharing’ was achieved (Greenwald & Shulman, 1973; Schumacher et al., 2001). Whether the absence of training-specific reductions in dual-task costs in the present study is related to robust capacity limitations in older participants (Sander et al., 2012) or to our specific training intervention is up for debate. Although our data cannot rule out the possibility of a ceiling effect, it is unlikely as the overall performance of the older adults is low compared to their young counterparts, even after having decreased from T0 to T1. Possibly, performance in highly demanding triple-task situations cannot be restored or compensated via training over and above the presented retest effects. In other working-memory paradigms, the age-related decline in cognitive performance was compensated by the recruitment of additional brain regions, such as right the lateral prefrontal cortex (Barulli & Stern, 2013; Heinzel et al., 2014) However, the present combination of cognitive dual tasks with postural demand might have exceeded the available capacity in old adults further, particularly in dual- (CC, CP) or (CCP) triple-task situations (Stelzel et al., 2017). Alternatively, the applied training intervention may have had some limitations that mitigated potential training responses: For example, participants trained in groups, which means that the supervising staff was limited in their ability to closely monitor individual execution of the exercises and correct errors. While group training can potentially incentivize participants to increase their work effort due to peers, it also allows for greater interaction between participants and may reduce their focus to perform the exercises at their best effort. Furthermore, participants did not receive any feedback regarding their performance of individual training sessions, giving them no opportunity to adapt their effort in relation to previous performance. Relatedly, physical and psychological effort of each session was solely based on subjective effort. As older adults have a tendency to underestimate their perceived level of exertion in relation to the true effort (Pincivero, 2011), it is possible that the use of objective biomarkers to assess training progress could have encouraged participants to increase their training level more regularly, thereby improving the efficacy of the intervention. Nevertheless, we focused on designing a training program that -if effective- could be disseminated and implemented in community or institution based settings to reach a large number of seniors in need of promoting their cognitive and motor performances. Therefore, we accepted the above described limitations when designing our intervention program.

The findings in regards of the effects of task load on postural control are consistent with results from previous experiments conducted by our research group (Stelzel et al., 2017). In general, participants demonstrated higher postural instability, indicated by greater COP displacements, when performing cognitive dual tasks (CCP) compared to cognitive single tasks (CP). Furthermore, older participants did not reduce postural sway in response to training but rather showed continuous increases in CoP path length in the CCP condition from T0 to T2 (***Figure 4***). The interference of cognitive-postural dual tasks in older adults is well-documented and has been linked to an increased risk of falls (Lundin-Olsson, Nyberg, & Gustafson, 1997). Effects are likely to be explained by theories on limited attentional resources (Kahneman, 1973). Within this framework, decreased balance performance is a result of interference that arises from performing concurrent tasks sharing the same attentional resources. Notably, the effects of task load were almost identical before and after the training, which raises the question whether age-related limitations in attentional resources can be modified by dual-task interventions.

The applied exploratory analysis for posture revealed a general reduction in cognitive task performance during semi-tandem stance compared to sitting. Moreover, cognitive dual-task effects were higher during standing. Given the greater demands on the postural control system, it is assumed that old adults exhibited cognitive performance declines due to age-related central resource limitations and larger attentional demands.

### Effects of Modality Compatibility Mappings

As expected, modality compatibility mappings affected cognitive performance in both training groups at baseline. The effects were most pronounced when two modality incompatible tasks were performed concurrently, which is in agreement with previous findings (Hazeltine et al., 2006; Stelzel et al., 2017; Stelzel et al., 2006). As modality compatible and incompatible dual tasks are identical in terms of perceptual and response processes, this dual-task specific effect has been related to crosstalk between central response-selection processes, differing between modality mappings. This crosstalk might involve the anticipation (Greenwald & Shulman, 1973; Hommel, 1998; Prinz, 1990) and monitoring (Wirth, Janczyk, & Kunde, 2018) of action effects. More recent evidence for this hypothesis has been provided by Schacherer and Hazeltine (2020) who directly manipulated the compatibility between stimulus and action effects and showed a modulation of dual-task costs in the expected direction. The observed persistence of greater dual-task costs for modality incompatible tasks compared to modality compatible tasks over the training period indicate the robustness of these effects in old adults. Manipulating the type and the salience of action effects in the context of a training intervention seems like a promising avenue for future research. Also, more explicit instructions of prioritization strategies might be a promising approach to increase the likelihood of achieving training effects further.

Surprisingly, we could not replicate our previous findings on detrimental effects of modality compatibility mappings on postural performance in the triple-task condition in the group of old adults (Stelzel et al., 2017). As the task design at baseline was widely identical to the one applied in this previous pilot study, this difference is difficult to explain. Maybe the general context of the experiment in this training sample, where participants were recruited for a balance training, led to a shift in prioritization in favor of the postural task, leaving less room for selective penetrability by specific cognitive demands. Also, the sample was more diverse, involving female and male participants, compared to a purely female sample in the pilot study, which might have led to greater variability in task performance. The analyses of individual differences indicate the necessity for multivariate approaches to investigate dynamic motor-cognition interactions with large sample sizes.

### Individual Differences

Notably, our data show large interindividual variability in response to the training protocols. Individual trajectories of cognitive and postural performance did not follow a clear pattern and suggest the presence of both responders as well as non-responders (***Figure 4***). Despite the fact that some participants (n = 8) exhibited overall gains in cognitive performance (4.57% ±2.31), we noted a considerable range (2.10%–7.43%) in the magnitude of training benefits. Furthermore, two participants demonstrated minimal changes (<±2%) in cognitive performance and eleven demonstrated a decline (–6.13% ± 2.85) after the intervention period. With respect to postural sway, six participants exhibited a decrease in CoP displacements (–11.07% ± 3.87) after the intervention, ranging from –5.7 to –16.34%. Four participants demonstrated minor changes (<±5%) and eleven participants increased their sway path (11.85% ± 7.54, range = 5.64%–29.92%) after training. The distribution of non-responders was similar across groups in both the cognitive (compatible training = 6, incompatible training = 5) and postural domain (compatible training = 9, incompatible training = 6), which suggests that the type of training did not have an effect on the heterogeneity of training responses.

Although we recruited participants that were similar with respect to age, health and mobility status and neuropsychology, we cannot rule out that variability in unknown subject characteristics affected the results and obscured possible training effects. Older adults vary greatly in their functional capacity, health status and physical capabilities due to diverging life trajectories (Dannefer, 1987) and as a result age-related heterogeneity is known to cause considerable variation in the receptiveness to training programs (Bouchard & Rankinen, 2001; Buford, Anton, Clark, Higgins, & Cooke, 2014; Chmelo et al., 2015). In order to investigate within-subject factors that may explain the large interindividual training responses, we conducted an exploratory analysis using PCA. However, the results provide limited explanation for our inconclusive results. Although individuals with higher attentional control benefited more from the intervention in terms of cognitive performance in modality incompatible conditions, the statistical relevance of these results is limited due to the small sample size.

Oddsson and colleagues (2007) argue that balance training interventions for older adults frequently disregard fundamental principles of training, such as continuity, overload, progression and specificity. We did our best to account for these issues in the design of our intervention protocol. The high adherence rates (97%) as well as the supervision of training sessions by qualified staff ensured that the training protocol was completed as intended, thus providing continuous stimuli for adaptation. Additionally, all sessions were documented by the staff, so that training levels could be increased according to objective and predefined criteria for each participant. These procedures ensured that participants were exposed to a gradual increase of cognitive and physical stimuli placed upon the body.

Overall, the highly heterogeneous training progress and the lack of a clear typical training response may indicate that our study cohort was too small and may have caused the observed non-significant outcomes following training. Due to several participants being excluded before the training and a high drop-out rate, a much smaller number of participants than suggested by an a priori power analysis was included in our dataset. This is particularly important, given the number of factors and the corresponding number of cells of our exploratory follow-up analyses. In this regard, a generalization of our findings to a broader aging population is not possible. In other words, our findings are always specific to the population under investigation. Nonetheless, the current experiment is valuable as it confirms existing research on the effects of dual-task training and it adds information to the existing body of research. We deem this study preliminary and it serves for future research on multitask balance training.

## Conclusions

The current study investigated the effects of cognitive-motor multitask interventions that differed with respect to compatibility of modality mappings on postural stability and cognitive performance in healthy older adults. In sum, our results support the view that the concurrent performance of cognitive and postural tasks is moderated by modality compatibility mappings, working memory load and increased postural demands. Contrary to our hypotheses, postural and cognitive performance as well as dual-task costs for both domains did not change in response to training. Instead, we observed increases in these parameters during a passive control period preceding the training intervention that were likely the result of repeated testing. Moreover, training protocols that differed with respect to compatibility of modality mappings failed to produce better results in congruent cognitive tasks. Exploratory analysis hints at the fact that individual differences in attentional control, working memory capacity and processing speed influence the prioritization of tasks in complex cognitive-motor multitasking situations, but given the low statistical power of these results, additional research is needed to confirm this assumption. Consequently, future studies should investigate interventions with different modality mappings in larger samples and include older adults that differ with respect to cognitive and mobility status. Findings of this study raise important questions regarding the effectiveness of multitask interventions for the purpose of improving cognitive-postural performance in older adults. Knowledge from this study will be helpful for designing and implementing future studies involving cognitive-postural multitask training.

## Data Accessibility Statement

The data that support the findings of this study are not publicly available due to data protection regulations.

## Figures and Tables

**Supplementary Figure:**
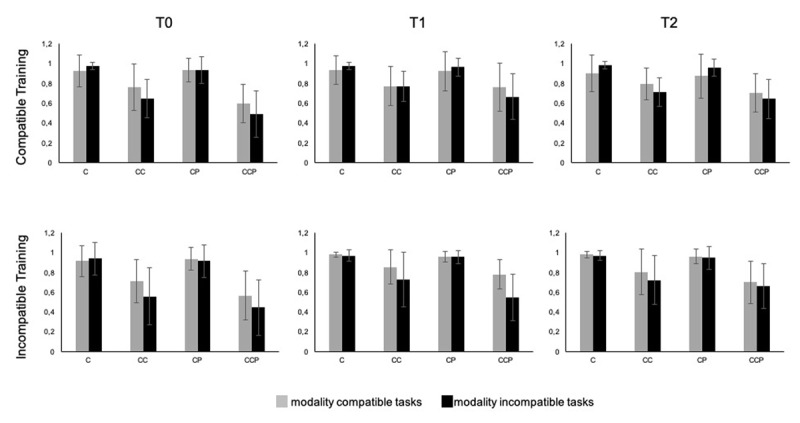
Mean (±SD) of cognitive performance (p(hit) – p(fa)) of both training groups for the different task types.

## Appendix

**Supplementary Table:** Correlation matrix for the PFA based on six neuropsychological items. (DTC_c_ = dual-task costs in the postural domain; TTC_p_ = triple-task costs in the postural domain).

VARIABLE		WORKING MEMORY	ATTENTIONAL CONTROL	PROCESSING SPEED

DTC_c_ Gain compatible	r	.264	–.146	–.308

	p	.260	.540	.187

	n	20	20	20

DTC_c_ Gain incompatible	r	.275	**.501***	.271

	p	.241	.024	.247

	n	20	20	20

TTC_p_ Gain compatible	r	**–.456***	.308	**.451***

	p	.043	.187	.046

	n	20	20	20

TTC_p_ Gain incompatible	r	–.286	–.253	.164

	p	.222	.281	.489

	n	20	20	20

* Significant at p ≤ .05.
